# On the Management of Twin Cases

**Published:** 1811-06

**Authors:** A. Fogo

**Affiliations:** Newcastle


*To the Editors of the Medical and Physical Journal.*

Gentlemen,

In your last Number, 146, there is what the writer calls "a singular case of twins," which terminated more favourably than happens in general under such treatment. The case is published from laudable motives, "that others may have an opportunity to pass their opinion on it." As the first child was "smaller than common," it is probable its membranes were weaker than common, and the adhesion of the placenta slighter than usual, and that premature labour was the effect. That the uterus being unloaded, had ceased to act for several days. It was a fortunate circumstance that the placentæ were not united, as is the case very often. Indeed if they had been united, the operator must have discovered the other child. The advice of the writer, under the circumstances he mentions, "the woman having continued well for the space of a week, without any dangerous symptoms," appears very judicious, and as the mouth of the womb was so much contracted, it was unnecessary to proceed to deliver. But this one singular case, and the practice followed in it, must not be looked on as a general rule in all cases of twins.

The

The writer complains that " the teachers of the present day are not sufficiently explicit in their directions for the management of cases, similar to the one I have related, namely, where the placentæ are separate, and each follows the body to which it belongs." It is not very likely that the public teachers of the present day ever heard of a twin being overlooked and left in the uterus for the space of six days. Whatever directions the modern teachers give, they could be of no use in the case, as the first gentleman not having discovered the second child, had no occasion for directions for what I suppose the writer means, whether to deliver or leave the affair to nature. The present case was left to nature, but as that was owing to a mistake or want of experience, it must not be considered as a precedent for our conduct. It may be said to be a termination more owing to good luck than good management. If the placentæ had been united, the result might have been very different.

The earliest writer on midwifery in my possession (Mauriceau) recommends the delivery of the second immediately after the first. He seems to have had as extensive practice as any of the moderns, and to all appearance as successful. He says, " Aussi-tot que j'eus tiré le premier dehors, je rompis les membranes des eaux du second, pour accelerer par ce moyen sa sortie ?" In twenty-eight cases of twins which he has published, he acted on these principles ; and it cannot be supposed he would have done so, if he had ever been unsuccessful, or observed any inconvenience or danger attending the practice. In fifteen of these cases the placentæ were joined ; the others were separated.

Dr. Burton, of York, also appears to have been in extensive practice, and gives the same advice. " The operator should introduce his hand immediately ; he must break the membranes without waiting for pains ; there will be room to turn the child if necessary, with ease to the operator, and with little pain to the woman ; after which it may be extracted by the feet."

Dr. Smellie is of the same opinion, and therefore it is unnecessary to transcribe from him.

Dr. Hamilton, as quoted by the writer, says, " In the interval (when the affair is left to nature) the patient is apt to suffer from impatience and anxiety. Floodings frequently come on, and the labour is more painful and hazardous, in proportion as the time of delivery is protracted. It may, therefore, be recommended to practitioners as a general rule, if labour pains do not naturally recur soon after the birth of the first child, to pass the hand gently into the uterus, break the membranes, and manage the delivery according to the presentation." Fifth edit. p. 383.

The Editor of the Edinburgh Practice of Physic (vol. v. p. 408) says, "the above practice is bad, whatever authors have said to the contrary;" but gives no reason for his opinion. He says, "If the woman is healthy, and the child presents favourably, natural pains ought to be waited for; (he does not tell us how long we ought to wait) when the child will be expelled by the force of these only: failing which, manual assistance must be had recourse to." Can an experienced or any practitioner get information from such advice? It is one among the many vacillating ambiguous expressions of no use to young or old.

Dr. Denman says, "I have concluded that we may safely and ought to wait for four hours at least, after the birth of the first child, before we deliver the patient by art of the second child, if there be no particular cause for delivering her sooner." (P. 401.) But in the former page he says, "*this (meaning the interval) from some cause we cannot comprehend or counteract, no efforts whatever are made for the expulsion of the second. This is a state of great solicitude to every person careful of his patient and of his own character, as he must know she will be in some degree liable to unpleasant, and even dangerous symptoms, till the second is also born, and the business compleated." Why then wait four hours at least, in a state of great solicitude to himself, and what is worse, of great danger to the woman and also the child, when the business may be safely compleated in four minutes? The above, like most of the directions in modern books of midwifery, is full of ambiguity, without giving a fixed or general rule by which a young practitioner can determine what conduct to follow.

The anonymous publication called The London Practice of Midwifery, (1803) said to be a copy of Dr. Clark's Lectures, speaking of twins and the method of delivering them, says, "Now with regard to the second child, deliver directly, says one; leave it to nature, says another; do nothing at all, says a third. We generally find truth in the medium. If we deliver directly we shall not be acting right; and if the woman is left to nature for two or three days, it is not right either. We must not leave her till she is delivered; if we

---

* We have not altered the text of our ingenious and respectable correspondent, but insert here the passage he quotes as it stands in Denman. (Introd. Midwif. 8vo. 2d Edit. vol 2. 399.) "But when after the birth of the first child, expelled in a reasonable time and by the natural efforts, from some cause which we cannot comprehend or counteract, no efforts whatever are made for the expulsion of the second child, the patient being as much at her ease as if there had been no previous labour; this is a state of great solicitude, &c." Editors.

do; do, we do it at her hazard. It is proper then to wait a moderate time." What is a moderate time? Here is the same indecision and ambiguity as mentioned by (I suppose his teacher Dr. Denman) the last author I have taken notice of; and which has no other effect than to puzzle and unhinge the young practitioner. He recommends waiting four hours as Dr. Denman has done, and subjects himself to great solicitude, and the woman and child to great danger. He then tells the young practitioner to amuse the woman with some insignificant chicanery, unbecoming a respectable professional man, to keep her in ignorance of there being another child, as he was advised by his teacher. But he goes on, "The time however comes when we see it necessary to turn and deliver, or at least to deliver. We have now to undo what we have been doing. To tell her the sponge (vid. p. 200) is separated, which it is very easy to set right without much pain." Could not the sponge have been set right without much pain in four minutes, by which he would have saved himself, the mother, her husband, and all concerned, four hours' anxiety, attended every moment with danger to both mother and child; and at last to surprise her with another child and two sponges.

The writer of the paper asks, "*If no unfavourable symptoms occur*, are we not justified to leave the second child to nature?" He thinks there is no danger from hæmorrhage unless the placentæ have been united. He is not sure that the part of the uterus to which the placenta was attached, may be pouring out blood and filling the space left by the child, which in an inactive uterus may not appear externally. As the case he has given, as I observed above, perhaps never before occurred, it must not be adopted as a precedent; and I hope such a case may never occur again: the fortunate termination of it must not be produced as a general rule in future cases of twins.

Dr. Denman (c. 17. s. 3.) says, "I have found the second child produced, or nearly so, before I had time to separate the first." He does not say there was any inconvenience or danger occurred to the woman or child from this rapid progress of the labour. It may then with great propriety be said, that if the second had not appeared so soon, there would have been no impropriety in proceeding to deliver the second as soon as he had discovered it, after disposing of the first.

Dr. Denman says, "by this decision (meaning waiting four hours) we shall certainly avoid many unnecessary operations, without detriment to the patient, without increasing our own difficulties, or hazarding our reputation." There cannot be many unnecessary operations; twins are not remarkably frequent; and as to delivering a second child, there is no difficulty that ever I experienced. But if hæmorrhage or other dangerous symptoms occur in the space of four hours, there will be detriment to the patient, and a serious hazard to the operator's reputation.

If any reader choose to look at page 234 et seq. of this London Practice of Midwifery, he will there see the result of a case left to nature, by the philosophising forbearing Dr. Hunter. I hope the tale is not true; if it is, he appears to have been guilty of little less than manslaughter; and I am suspicious that his doctrines, infused into thousands, are the cause of the forbearing system, and that of leaving every thing to nature in the present fashionable practice.

There is not the shadow of a reason in the London Practice of Midwifery, or in any other book I have seen, why we should not deliver the second child soon after the first. The writer talks as his teacher (I suppose) of waiting four hours. "We should therefore let her have an hour to cool and to nourish; and if when she begins to feel herself strong, she begins to be again uneasy about the after-birth, we may alarm her a little. So after playing this piece of artifice, she will be quiet enough?" But if the woman has not been heated or fatigued, from having had a moderate easy labour, why wait four hours? Granting she has been fatigued and heated, during the extraction of the second child, she lies quite passive, undergoing no additional exertion; and the extraction of the child and placentas must abstract a very considerable quantity of caloric from her system, by which she will be cooled much sooner than she would have been undelivered. The natural discharge from the uterus may also contribute to assist in cooling her.

I have now mentioned the practice I have adopted through a long life; it has been uniformly successful in cases of twins. I hope the professor you allude to will speak as decidedly, whether for or against me; and that he will produce proof where he has seen inconvenience or danger occur from the immediate delivery of the second child, whether natural or cautiously artificial.

I conclude by relating, in few words, a case I was informed of by the midwife. The woman bore a child on the Sunday morning. No pain after:—on the Monday she began to be alarmed, finding there was another child, and sent for a surgeon, who did nothing, but desired he might be sent for if pains recurred. On Tuesday evening slight pains, with no advantage. The mother and midwife, M. W. were sensible the child was alive and very active. The surgeon, who had had been again called on the appearance of pains, proposed to take some blood from the arm, which was submitted to with great reluctance, the patient never having been bled before; and had a great dread of the operation. She returned to bed, and in a short time told the midwife that she had not felt the child move since the bleeding. On Wednesday evening the pains recurred stronger; the surgeon was sent for, but before he arrived another child was born dead; and soon after that, a third, dead also.

Here was a case left to nature, though not entirely, terminating in the loss of two full-sized boys, which, in my humble opinion, might and ought to have been extracted on the Sunday morning.

I am, Gentlemen,

Your most obedient,

A. FOGO.

*Newcastle, April* 11, 1811.

